# What does it take to provide clinical interventions with temporal consistency? A qualitative study of London hyperacute stroke units

**DOI:** 10.1136/bmjopen-2018-025367

**Published:** 2019-11-07

**Authors:** Georgia B Black, Angus I G Ramsay, Abigail Baim-Lance, Jeannie Eng, Mariya Melnychuk, Penny Xanthopoulou, Martin M Brown, Stephen Morris, Anthony G Rudd, Robert Simister, Naomi J Fulop

**Affiliations:** 1 Department of Applied Health Research, University College London, London, UK; 2 City University of New York System, New York City, New York, USA; 3 Barts Health NHS Trust, London, UK; 4 Department of Applied Health Research, Imperial College London, London, UK; 5 Department of Psychology, University of Exeter, Exeter, UK; 6 Department of Neurology, University College London, London, UK; 7 Clinical Effectiveness and Evaluation Unit, Royal College of Physicians, London, UK; 8 Comprehensive Stroke Service, University College London Hospitals NHS Foundation Trust, London, UK

**Keywords:** stroke, organisation of health services, qualitative research, health policy, stroke medicine

## Abstract

**Objectives:**

Seven-day working in hospitals is a current priority of international health research and policy. Previous research has shown variability in delivering evidence-based clinical interventions across different times of day and week. We aimed to identify factors influencing such variations in London hyperacute stroke units (HASUs).

**Design:**

Interview and observation study to explain patterns of variation in delivery and outcomes of care described in a quantitative partner paper (Melnychuk *et al*).

**Setting:**

Eight HASUs in London.

**Participants:**

We interviewed HASU staff (n=76), including doctors, nurses, therapists and administrators. We also conducted non-participant observations of delivery of care at different times of the day and week (n=45; ~102 hours). We analysed the data for thematic content relating to the ability of staff to provide evidence-based interventions consistently at different times of the day and week.

**Results:**

Staff were able to deliver ‘front door’ interventions consistently by taking on additional responsibilities out of hours (eg, deciding eligibility for thrombolysis); creating continuities between day and night (through, eg, governance processes and staggering rotas); building trusting relationships with, eg, Radiology and Emergency Departments and staff prioritisation of ‘front door’ interventions. Variations by time of day resulted from reduced staffing in HASUs and elsewhere in hospitals in the evenings and at the weekend. Variations by day of week (eg, weekend effect) resulted from lack of therapy input and difficulties repatriating patients at weekends, and associated increases in pressure on Fridays and Mondays.

**Conclusions:**

Evidence-based service standards can facilitate 7-day working in acute stroke services. Standards should ensure that the capacity and capabilities required for ‘front door’ interventions are available 24/7, while other services, for example, therapies are available every day of the week. The impact of standards is influenced by interdependencies between HASUs, other hospital services and social services.

**Strengths and limitations of this study**

- This is the first study to observe 7-day working practices to understand how temporal variation and consistency in clinical interventions is created.
- We interviewed a wide range of clinical professions to build a diverse picture of hospital practices; a limitation is that we did not interview all relevant professionals, especially outside the hyperacute stroke unit (HASU).
- We observed HASUs and other areas of the hospital at different times of day and night to see what is done differently.
- Our paper is partnered with a statistical analysis of temporal variations in patient admission, delivery of evidence-based interventions and outcomes, creating a full mixed methods approach.
- This study was focused on London’s HASUs: lessons may not apply to all hospital contexts.

## Introduction

Seven-day provision of consistent, high-quality urgent and emergency care settings is an international research and policy priority.^1–6^ This is motivated by 15 years of attention placed on the enigmatic ‘weekend effect’ and other temporal variations,^7^ scrutinising mortality and other outcomes for those admitted at the weekend compared with during the week.^5 8–14^ Varying features that may explain differential outcomes include staffing levels^8^ and patient mix,^15^ leading to widespread interest in studying weekend organisational features.^7 16^

For urgent stroke care, recent studies have shown that temporal variation in mortality is now decreasing^17 18^ including in London’s centralised ‘hub and spoke’ model^19^ and in the USA ‘comprehensive stroke centres’.^6^ London has eight ‘hub’ units which are designated as hyperacute stroke units (HASUs). These units were opened in 2010 following a reorganisation of the London stroke model to create a small number of 24-hour acute assessment and treatment centres (the ‘hub’) linked to a network of stroke units across the city (the ‘spokes’) capable of receiving patients from the HASU for ongoing stroke care. This reorganisation of care has attracted interest through significant improvements in evidence-based care^20^ and greater reductions in mortality^21^compared with the rest of England. All eight HASUs are subject to the same service specifications with respect to staffing levels of key groups, access to imaging and access to dedicated stroke beds and all are required to provide a 24-hour service capable of rapid assessment by a stroke team, early treatment using thrombolysis (clot-busting drugs) if needed, high-dependency monitoring and a 24-hour specialist team.^22 23^ At the time of our study, no HASU was providing a formal thrombectomy service for stroke associated with large vessel occlusion, but currently three of the eight HASUs provide 7-day thrombectomy services and one of these is a 24-hour service. Further reorganisation of the London model is currently in progress with the designation of a subset of HASUs that can can provide centralised thrombectomy. The model is governed by the London Stroke Strategy which imposes tariff-linked requirements for staff level consistency and sufficient evidence-based care at all times; this strategy may have facilitated better outcomes.^22^ We show in a companion paper^24^ that in the London HASUs, there was:

1. No variation by day/time of admission in 3-day mortality or disability at hospital discharge.
2. No variation by day/time of admission in delivery of ‘front door’ interventions, such as stroke nursing assessment, brain scanning and thrombolysis measures.
3. Significant variations by day/time of admission in other interventions (including timely consultations and assessments with stroke specialists and therapies, and length of hospital stay).

In this paper, we set out to explore *how* acute care interventions are delivered with temporal consistency in centralised acute stroke services, and *why* some care interventions are resistant to temporal consistency by examining the organisation of services at different times of day/week.

## Methods

Our methods are reported according to Consolidated criteria for Reporting Qualitative Research reporting guidelines.^25^

### Recruitment and sample

Authors GBB, JE, PX and AB-L conducted 76 interviews with HASU staff and 45 non-participant observations of HASU activity over the day, night, week day and weekend (including HASU team meetings and care provision across the acute stroke pathway) over approximately 102 hours. GBB, PX and AB-L have doctoral degrees, extensive interview training and research experience. JE was trained in interviewing for this study and has a background in stroke occupational therapy. Thus, the team had ‘insider’ knowledge used to interpret behaviours and build rapport, but also ‘naive’ outsider perspective used to ask taken-for-granted questions of the staff. JE conducted informal discussions with ward managers from each site to establish staff numbers and seniority on the ward, the composition of staff attending suspected stroke calls in the emergency department (ED) and the availability of scans across the week.

### Non-participant observations

We conducted non-participant observations at least four times at each HASU site: two visits in the weekday, one in the evening during the week and one at the weekend, with additional visits to confirm or add to our findings. We collected data on various aspects of HASU activity likely to influence care provision, guided by clinical interventions in our quantitative analysis, and our initial observations (see table 1).

**Table 1:** Summary of activities observed during non-participant observations

Activities observed	Total (of eight sites)
‘Front door’ activity	7/8
Ward round	6/8
Multidisciplinary team	7/8
16:00 catch up meeting	3/4*
Nurse handover	8/8
Bed meeting	6/8
Discharge	4/8
Total conducted	41

Purposive sampling of observations is described under Methods section.

*Only four hyperacute stroke units have this activity.

We initially structured observations so that availability of staff and key processes (eg, handover and ward rounds) were covered. Subsequently we targeted our observations toward our emerging analysis, such as following patients’ journeys from the ED to the HASU ward, and shadowing the nurse-in-charge and consultants to understand their roles.

We gained global written consent for observations from service leads and from a selection of staff at team meetings. Thereafter, verbal consent was given by staff members for researchers to make observations of staff meetings and of care provision.

#### Interviews

Staff were sampled purposively to ensure coverage of a range of professional roles within all eight HASUs, with perspectives on the clinical interventions analysed in our accompanying paper in this issue^24^ (eg, interpreting brain scans, thrombolysis, therapy assessments) including medical, nursing, therapy and administrative or managerial staff (table 2).

**Table 2:** Summary of interviewees

Profession	n
Consultant physician*	11
Junior doctor†	15
Senior nurse‡	7
Stroke nurse	8
Research nurse	1
Physiotherapist	10
Occupational therapist	8
Speech and language therapist	8
Stroke coordinator§	8
Total conducted	76

*Includes lead consultants.

†Includes specialist registrars and senior house officers.

‡Includes matrons and ward managers.

§Includes stroke coordinators, facilitators and administrative staff.

Researchers approached staff during the observation work and through existing contacts with study information, stating that participation would be confidential and anonymously presented in publications. Interviews were conducted with fully informed, written consent and in private settings, according to a semistructured topic guide (online supplementary file A) with the aim of understanding temporal variations from the staff perspective, including typical daily activities and attitudes toward working in and out of hours. Interviews lasted between 20 min and an hour. No field notes were taken, but each interview was discussed with the other team members. Interviews were audio recorded and transcribed verbatim.

10.1136/bmjopen-2018-025367.supp1 Supplementary data

### Analysis

Quality and trustworthiness were maintained through a select number of joint observations and interviews to sensitise ourselves to pertinent contextual issues, and regular reflexive discussions between the researchers. We analysed data in three phases, following the methodological principles of inductive/deductive thematic analysis.^26^ First, four researchers (GBB, AB-L, JE, AGR) developed codes through an analysis of 20 interview transcripts, using independent and joint coding to develop inter-rater consistency, and these codes were then discussed with the wider research team. Second, we performed further deductive coding of the remaining data using a broad coding framework developed in response to the research questions. We used Microsoft Word and Excel to manage the data. The third phase developed iteratively by group interpretation of the coded data, focusing on the research questions and unexpected findings. Finally, we mapped the results against the variations in practice across the 7-day week found in our analysis of the performance data for care provision in London HASUs set out in Melnychuk *et al*,^24^ in particular the distinction between ‘front door’ clinical interventions (which were provided consistently) and other interventions (which were not).

### Patient and public involvement

Two stroke patient representatives contributed to our study protocol, research questions and discussions of interim findings presented at steering committee meetings in June 2015 and July 2016. They raised issues related to staff handover and confirmed the importance of the interface between hospital and social services, which we incorporated into our analysis.

## Results

London HASUs deliver ‘front door’ clinical interventions, including brain scans, thrombolysis and swallow assessment without significant temporal variation.^24^ Length of stay showed significant variation without a clear temporal trend, assessment by a stroke physician within 12 or 24 hours varied by time of day and assessments by therapists varied by the day of the week. Care quality was worse for patients admitted on a Friday.

In the following sections, we present: (1) factors that may explain why ‘front door’ interventions showed temporal consistency, as well as some unintended consequences of the strategies London HASUs employed and (2) factors that may explain why other interventions and outcomes show significant temporal variations (table 3).

**Table 3:** Themes as they relate to consistently and inconsistently provided clinical interventions

	Theme	Impact
Factors influencing temporally consistent care in nursing assessments, CT scans and thrombolysis	Adapting and extending roles	At night the consultant was only called for positive confirmation if the registrar thought a patient eligible for thrombolysis.
	Creating continuities between different times of day	HASU staff created continuities between team members operating at different times of day/week. Handover meetings, multidisciplinary team meetings and ward rounds.
	Building relationships and trust	Strong, trusting relationships with, eg, ED and neuroradiology staff. Reduced decision-making delays, helped maintain pace of assessment and delivery clinical interventions (especially when more than one patient in ED).
	Prioritisation of ‘front door’ interventions	HASU staff relished the early stages of acute stroke care and the potential to see rapid positive outcomes.
	Unintended consequences of adaptations	Threshold for admission to the HASU weakened (junior doctors more risk averse)—led to greater pressure on beds.
Factors influencing temporally inconsistent care in ward admissions, consultant assessments in 12 and 24 hours and therapy assessments in 72 hours	Variations in medical, managerial and allied health professional staffing by time of day	Likelihood of admission to HASU within 4 hours was influenced by the number of potential patients arriving at hospital. Undergoing consultant assessment within 12 hours and 24 hours depending on patients reaching the ward during period 09:00–12:00.
	Variations in delivering therapist assessments	Therapists worked ‘in hours’ shifts—patients arriving at hospital in the morning were less likely to be assessed within 24 hours unless assessed on the day of arrival. London standards specified therapy staffing levels to fully cover only 5 days per week. Various attempts to cover weekend working, but no current staffing model permits consistent therapy provision.
	Variations in repatriation processes	Patients admitted at weekends less likely to be seen by therapists. Social services, care homes, stroke units and community rehabilitation units were significantly less likely to accept new cases at weekends.

ED, emergency department; HASU, hyperacute stroke unit.

### Factors influencing temporally consistent care

**Figure 1:**
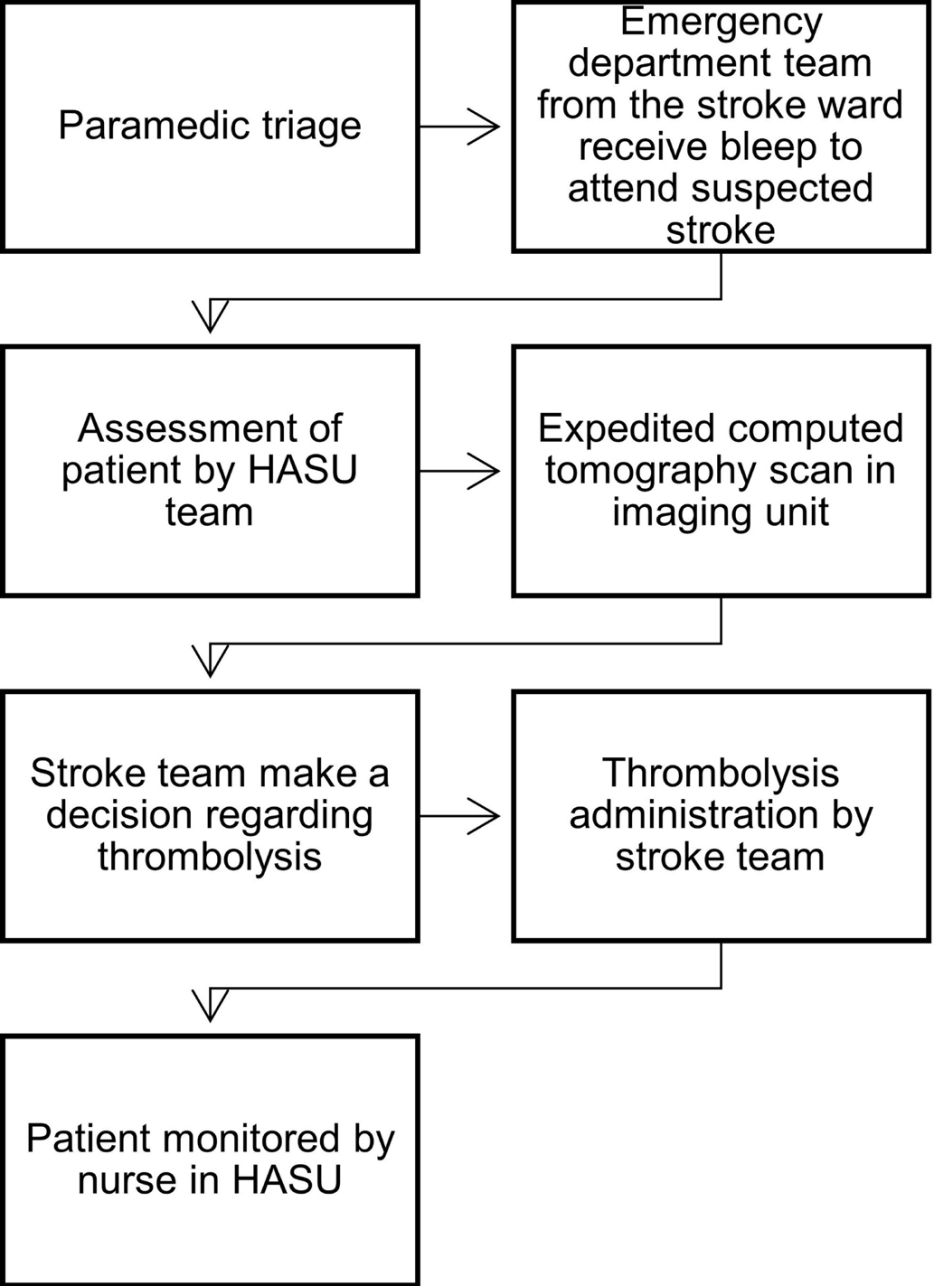
Thrombolysis pathway (adapted from Catangui and Slark^29^). HASU, hyperacute stroke unit.

The London HASU standards required that HASUs have 24/7 availability of staff who are trained to assess eligibility for and deliver thrombolysis; this was normally completed by a team of medical and nursing staff who are on stand-by to immediately attend patients at ED when alerted to new potential patients with thrombolysis by the ED team (see figure 1).

**Figure 2:**
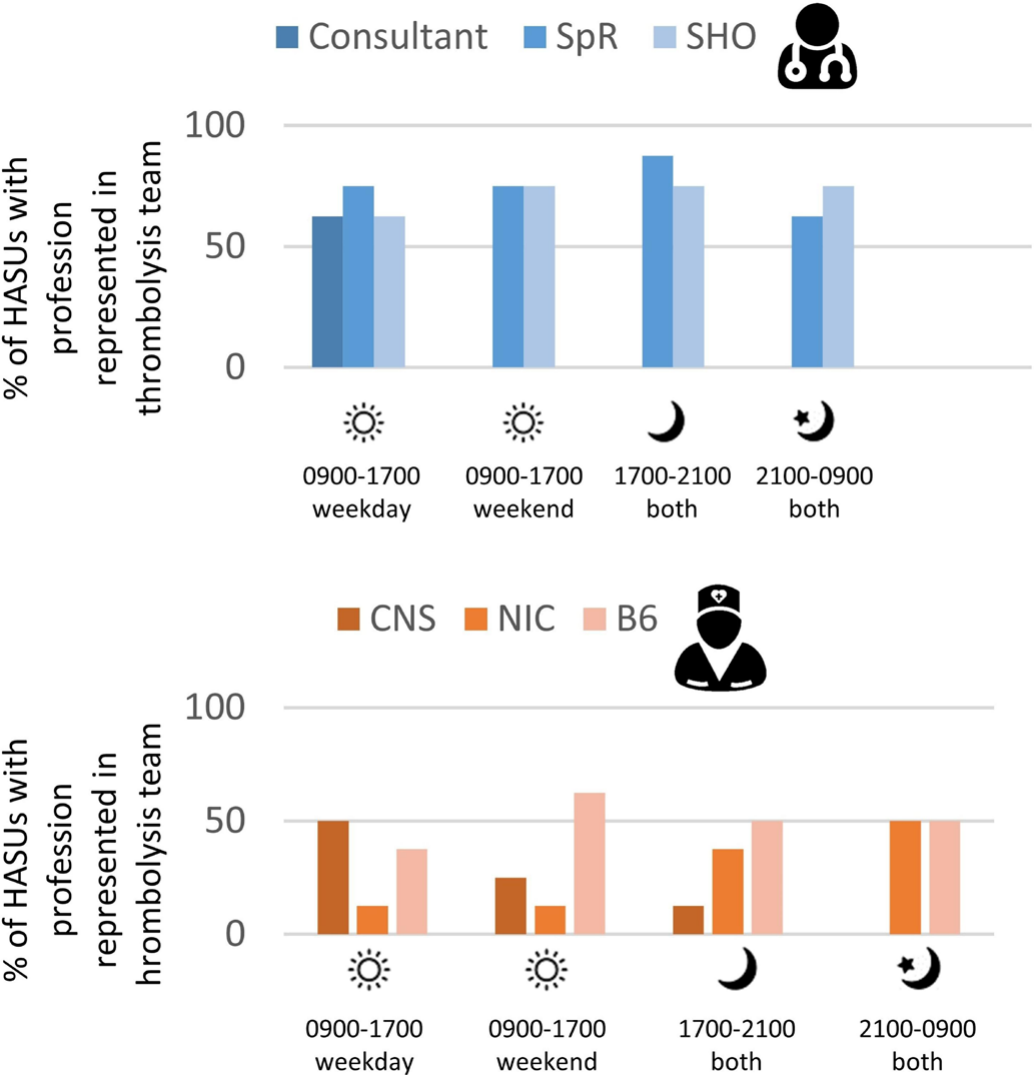
Staff attending suspected stroke calls in emergency department. B6, band 6; CNS, clinical  nurse specialist; HASUs, hyperacute stroke units; NHS, National Health Service; NIC, nurse-in-charge; SHO, senior house officer; SpR, specialist registrar. Notes: Banding refers to NHS standardised pay grades and increases according to seniority. Staffing may have altered since the time that this information was collected.

Thrombolysis was administered by the stroke team where appropriate, after a battery of clinical and imaging tests had been conducted. The time taken to complete this process is one of the core performance metrics by which each treating unit is measured. Each HASU attempted to make this pathway as efficient as possible having a designated ‘thrombolysis team’ irrespective of clinical activity elsewhere in the stroke service and through local arrangements with radiology to guarantee that 100% of patients with stroke potentially eligible for thrombolysis are scanned within the next CT scan slot. However, as detailed in figure 2, the staff supporting ‘front door’ activity reduced both in number and seniority in the evenings and on weekends. As a result, the HASU teams made significant adaptations out of hours, including extending roles and responsibilities, and introducing processes to ensure continuity between different times of day.

### Adapting and extending roles

At all times of day, stroke specialist registrars and senior house officers (junior doctors) made decisions about thrombolysis with consultant telephone support. However, observations and interviews suggested that in some HASUs at night, the consultant was only called for positive confirmation if the registrar thought a patient eligible for thrombolysis (for additional data, see online supplementary file B, section 1a). This was seen as a positive educational opportunity for junior doctors, but it placed greater responsibility on senior nurses to decide whether or not to admit the patient to the HASU in cases where the stroke diagnosis was unclear.

10.1136/bmjopen-2018-025367.supp2 Supplementary data

#### Creating continuities between different times of day

Rapid movement of patients through the hyperacute stroke pathway required quick resolution of problems, and transfer of detailed information between team members who work at different times of day/week (see online supplementary file B, section 1b). Key mechanisms took the form of handover meetings, multidisciplinary team meetings and ward rounds:

Bay nurse leads on his patients, going through the discharge sheet that everyone has and focuses on any particular issues that have arisen, or that the next nurse needs to be aware of. (Evening observation, H4)

Other ways of creating continuity included shifting or staggering staff rotas (to bridge gaps between shifts), and extending therapists’ hours into the early evening.

#### Building relationships and trust

Delivering front door interventions consistently depended on rapid decision-making, an important facilitator of which was the development of strong, trusting relationships with allied disciplines, in particular ED and neuroradiology (see online supplementary file B, section 1c).

HASU staff worked alongside ED staff to assess the patient, and there were often overlaps when a patient’s diagnosis was unclear, or when multiple patients presented at once. HASU staff felt that ED clinicians also valued this close relationship:

We are popular with the ED team […] we are one of the few teams where you’ve got a consultant down there sweating away with them. (Consultant physician, H2)

HASU staff also had an important relationship with radiology, with prioritised access to CT scans as outlined in the London stroke service standards. The HASU team often conducted initial interpretation of CT scans, which reduced decision-making delays. HASU staff felt that this self-sufficiency strengthened their relationship with ED and radiology staff.

#### Prioritisation of ‘front door’ interventions by staff

HASU staff’s enthusiasm was an important facilitator of sustained performance in delivering ‘front door’ interventions (see online supplementary file B, section 1d). Interviews and observations (eg, of the urgency with which staff responded to calls to attend ED) suggested HASU staff relished the early stages of acute stroke care and the potential to see rapid positive outcomes:

When the stroke happens we have to work fast: run to ED, do everything within four hours to ensure that the patient can be thrombolysed, and I’ve seen the patient like almost dead […] we were able to save the patient’s life because immediately we were able to assess, go for CT, thrombolyse the patient […] to me it’s great work. (Stroke nurse, H2)

#### Unintended consequences of adaptations

While extending roles out of hours was seen as an opportunity for staff development, some interviewees felt the threshold for admission to the HASU was weakened (see online supplementary file B, section 1e). Junior doctors were seen as more risk-averse than consultants, thus admitting more patients unnecessarily out of hours, in turn placing greater strain on the service at a time when it is particularly difficult to move patients from the HASU:

Some doctors, they will send them in to you, put the pressure on you to take that patient […] in the morning when the consultant sees the patient, this patient has not had a stroke […] once the patient gets in here, it’s difficult to send the patient back to the wards, that becomes a big problem, they’re here 1 week, they’re still waiting for medics to take the patient over. (Senior nurse, H1)

### Factors influencing temporally inconsistent care

Variations in outcomes and delivery of clinical interventions in London HASUs are summarised in table 4. We explain these findings in terms of reductions in both number and seniority of medical, managerial and allied health professionals out of hours, and reductions in repatriation options out of hours.

**Table 4:** Summary of variations in care and outcomes identified by Melnychuk *et al*.^24^

Type of variation	Examples
Time of day but not day of the week	Admission to hyperacute stroke units within 4 hours Most likely: arriving at hospital 00:00–04:00 Least likely: arriving at hospital 08:00–17:00 Assessment by a stroke consultant within 12 hours Most likely: arriving at hospital 00:00–04:00 Least likely: arriving at hospital 16:00–20:00 Assessment by a stroke consultant within 24 hours Most likely: arriving at hospital 16:00–20:00 Least likely: arriving at hospital 04:00–08:00
Day of the week but not time of day	Therapist (Physiotherapist, Occupational Therapist, Speech and Language Therapist) assessments within 72 hours Patients admitted on Friday less likely to be assessed
Time of day and day of the week	Therapist assessments within 24 hours Variation during the day Monday–Friday (least likely arriving at hospital 04:00–12:00) Patients admitted on weekends less likely to be assessed
Outcome	Length of stay Longer for patients admitted at weekends

#### Variations in medical, managerial and allied health professionals by time of day

Variations in delivery of interventions by time of day but not day of week (table 4) are likely to have been influenced by variations in HASU activity and staffing variations that derive from the London HASU standards (see online supplementary file B, section 2a).

While some staffing levels were lower at night, the number of patients arriving also reduced, giving patients a better chance of being admitted the HASU within 4 hours:

We know less people have strokes overnight […] so we know it’s like going to be quieter, but from a staffing perspective, that’s why we’ve done the 24 hours thing, so there is always those amount of staff on. (Senior nurse, H4)

Patients were assessed during the consultant-led ward round, which the standards required to take place daily and which commonly occurred between 09:00 and 12:00. Undergoing consultant assessment within 12 hours and 24 hours thus depended on patients reaching the ward during this period. For example, if a patient arrived at 03:00, their first consultant assessment would be likely to take place during that morning’s ward round (~6 hour wait); if they arrived at 15:00, it would be likely to occur the following morning (~18 hour wait):

After the ward round has finished and we’ve tidied up a bit, yes, you’re less likely to come back and see a case, unless it was very urgent or some unusual type thing. (Consultant physician, H4)

#### Variations in delivering therapist assessments

Therapists generally worked ‘in-hours’ shifts, so patients arriving at hospital in the morning were unlikely to be assessed until the next day (see table 4), in the morning after a board round where the team make daily decisions about individual patients’ care. This meant that patients arriving at hospital between 04:00 and 12:00 were the least likely to be assessed within 24 hours.

The London standards specified therapy staffing levels to fully cover only 5 days per week, and thus HASUs faced a decision on how best to use these limited  resources; some chose Monday to Friday because of traditional working patterns on these days (see online supplementary file B, section 2b). The resultant gap in therapist coverage at weekends explained why patients admitted on a Friday were less likely to undergo therapy interventions within 72 hours of arrival (whereas patients admitted on the weekend were more likely to be assessed on the following Monday or Tuesday). Therapists described feeling rushed on a Friday as they struggled to get through their workload before the weekend:

Yeah I think it can be very stressful on a Friday, just if patients are going home when there’ve been a few discharges at the same time it can get quite complicated trying to coordinate a lot of family members, patients, staff to fill in documentation, social work, making sure a care package has gone in and completing lots of referral forms. (Speech and language therapist, H2)

Other HASUs spread their limited therapy resources into the weekend, which was reported to have a beneficial outcome on discharge figures, but participants reported a change in priorities. Assessment of new patients dominated, and therapeutic work or talking to families was diminished. Further, by spreading therapist resources into the weekend HASUs reduced therapist capacity during the week. Therapists in almost all HASUs suggested that existing attempts to cover weekend working resulted in reduced prioritisation of therapeutic activity, suggesting no current staffing model permits consistently sufficient therapy provision.

#### Factors influencing length of stay

Patients admitted at the weekend in London had a greater length of hospital stay. This related to a number of factors (see online supplementary file B, section 2c). As patients admitted at weekends were less likely to be seen by therapists, this resulted in patients not having their rehabilitation and nutritional needs potentially for 3 days in a row:

If you’re nil by mouth on the Friday when you come in, say at half past four … you could technically be nil by mouth until Monday. (Occupational therapist, H1)

Reduced therapeutic capacity could also delay discharge, thus extending length of stay:

you think, ‘Well, this person can’t go because they need a Physio, and we could have discharged them on the Saturday but they have to wait till Sunday or even Monday,’ which […] can cause a problem sometimes if we need beds […] and that person’s then spent another day potentially in hospital that they potentially don’t need to (Occupational therapist, H8)

HASU staff suggested that social services, care homes, stroke units (acute rehabilitation units) and community rehabilitation units were significantly less likely to accept new cases at weekends. This restricted the timing of discharges from the HASU, often leading to longer stays. Input from social services and Early Supported Discharge (a service designed to accelerate the discharge home of patients in hospital^27^) was important in ensuring patients returned home or to care homes quickly once sufficiently recovered. However, social services have extremely limited weekend operation, which prevents liaison during the weekends to prepare packages of care or transfer patients (online supplementary file B, section 2c).

## Discussion

To our knowledge, this is the first qualitative study about the organisation of stroke care with respect to temporal variation. This study reports qualitative data that help explain the findings presented in Melnychuk *et al*.^24^ Consistent provision of clinical interventions was underpinned by: (1) junior nursing and medical staff extending their in-hours responsibilities to cover key decision-making roles, such as that of the thrombolysis nurse; (2) intervening to bridge potential gaps caused by shift-working (staggering rotas, holding meetings to share information) and (3) HASU leadership building trust and respect across staff both within HASUs and within key specialties elsewhere in the hospital (such as ED and neuroradiology). Key issues leading to temporal variation in care provision included reductions in medical, managerial and allied health professions, and significantly reduced options for repatriation to other acute services and community services, at night and on the weekend. Variations resulted in greater pressure on the ward from low thresholds for admission at night, dilution of staff capacity and bottlenecks in repatriation pathways. Some of these effects were mitigated by strategies to create ward space and expedite discharges on a Friday, but these strategies had a number of unintended negative consequences in terms of patient outcomes.

The London service standards were an important influence on delivery of clinical interventions, whether consistent or inconsistent. Where standards required 24/7 availability of staff, for example, nurses, aspects of care associated with these staff groups tended to be delivered consistently, regardless of time of day or day of week. Where standards required that a key activity was conducted on a daily basis (such as the consultant-led ward round), the likelihood of patients undergoing the associated intervention varied significantly according to when they were admitted. Finally, where standards specified staffing levels to cover only 5 days (as with therapies), it was not possible to provide interventions consistently over 7 days, regardless of local adaptations employed.

The strengths of our study are founded on detailed data collection in each London HASU at different times of day both during the week and at weekends, providing a rich picture of the realities of organising and providing a high-performing acute care system. Observing and comparing how eight sites organised themselves in different ways to meet the same standards affords generalisability to our results. There were several limitations to the study. First, we did not study any hyperacute stroke services operating within a different service model (whether centralised or non-centralised). The lack of a comparator limited our confidence that our findings explain 24/7 care per se (as compared with a centralised model of care) with respect to the quantitative analyses in Melnychuk *et al*.^24^ Second, we did not interview all relevant professions within the studied organisations, for example, pharmacy, emergency medical practitioners and so on. Therefore, our perspective on important working relationships beyond the HASU was based on HASU staff perceptions, though they were supported by our own observations particularly of ED coordination. Finally, these services develop constantly, and some aspects of provision such as staffing levels are likely to have changed.

### Recommendations for research, policy and practice

This study adds to current knowledge as the first qualitative study to provide explanations for how and why temporal variation arises in stroke, and how it can be mediated. Our study was strongly in accord with the growing body of literature suggesting that different patterns of temporal variation are relevant to specific clinical interventions and outcomes.^7 8 19^ Clinical decision-makers looking to improve temporal consistency in stroke care should consider different weekend therapy working patterns and extended working hours for all clinical disciplines. However, managers should be cautioned that without increased resource, bottlenecks in workload are caused by reduced staffing and repatriation options at weekends, placing staff under strain and deprioritising non-urgent patients. Our findings are relevant internationally with respect to reducing temporal variability in stroke outcomes,^5 6^ and in other acute care settings.^4 28^

Policy makers and clinical decision-makers promoting 7-day health services should apply clear service standards, which facilitate the delivery of clinical interventions. The standards should consider how each health profession might contribute to 7-day care, at what time of day, and what capacity is required to deliver this. Standards must recognise how these will influence patient flow, and acknowledge service interdependencies both within and beyond the hospital perimeter. Multidisciplinary evaluations of efforts to provide 7-day care such as this can help planners avoid unintended consequences of service reorganisations, both in terms of gaps in the models implemented and how clinical teams respond to these.

Researchers need to examine other efforts to deliver clinical interventions 24/7 in stroke and other clinical settings. In-depth analysis of the interdependencies that influence 24/7 delivery of care, both within and beyond the host hospital, would be of value.
