# Supplementary material for: What does it take to provide clinical interventions with temporal consistency? A qualitative study of London hyperacute stroke units
Source: BMJ Open. 2019 Nov 7;9(11):e025367. doi: 10.1136/bmjopen-2018-025367 (PMC6858131; doi:10.1136/bmjopen-2018-025367)
Supplement: Supplementary data [file bmjopen-2018-025367supp001.pdf]

HASU 24/7 study: Staff interview guide

Thank you for agreeing to take part in this interview. I'm really looking forward to hearing your views on how things work.

The purpose of the interview today is to get your views of the Hyperacute Stroke System and specifically on the 24/7 working.

I'll try to keep things open ended so that you can expand on the issues that you think are important. It goes without saying that there are no 'right' or 'wrong' answers. The interview transcript will be anonymised, ensuring you cannot be identified and nothing will be attributed directly to you or to your team.

Any questions before we start?

Practicalities

- Ask to sign consent form (x2), Confirm audio-recording, Record staff interview ID, Transcript required?

Interviewee's background/role

1. How long have you been a member of this HASU for?
2. What would you describe as your primary role at the HASU? (Any other roles?)
3. What is your experience of stroke & how long (years/months)?
4. What shift do you usually work? What shifts have you previously worked?
5. How do you find working in Stroke? Do you plan to continue working in this area/specialise?

Processes

6. How are staffing levels decided and organised IH & OH?
    a) Is the staffing/staff expertise appropriate in relation to the workload
    b) Impact on patient care/experience/continuity of care/staff (sickness, reduced productivity, staff turnover)

7. Thinking about processes to support quality care, such as MDT meetings, have any groups been set up to oversee this (both within the service and across the whole system)?
    a) Participation/ engagement
    b) Supervision/training & support
    c) Audit/Data collection
    d) Impact on staff/patient care/workload

Delivery of HASU care

8. How are patient admissions at the HASU organised and delivered IH and OH?
    a) Is patient and carer experience the same independent of the admission time?
    b) Do staff dealing with admission IH and OH have the same level of expertise?

9. In terms of access to supporting investigations, how are these delivered IH & OH?
    a) Are scans and tests available and delivered by the same staff?
    b) Impact on patient experience and care/staff?

10. How are treatments and therapies delivered IH and OH?
    a) Information, dignity, accessing consultant/nurse/therapist/workload
    b) What would you do if a patient deteriorates IH/OH?/Impact on staff/patients

11. Do you think patient and carers receive enough information IH /OH and when they leave the HASU?
    a) Impact/Carers' experience?

12. How is communication across the whole system (IH vs. OH)?
    a) Within the hospital & beyond the hospital?
    b) How different professional groups interact? What is the morale like?
    c) Repatriation /Handover?
    d) Impact on patient experience/continuity of care/workload?

13. Overall what are obstacles and enablers to delivering 24/7 working?

Concluding questions

14. Has anything changed about the way this HASU operates? Since its establishment/since last year/a month ago?
    a) What drives changes? (local/national policy, staff/patient needs/issues, targets)
    b) In what ways you and your colleagues would like things to change?

15. Finally, is there anything else that you'd like to add?
