# Supplementary material for: What does it take to provide clinical interventions with temporal consistency? A qualitative study of London hyperacute stroke units
Source: BMJ Open. 2019 Nov 7;9(11):e025367. doi: 10.1136/bmjopen-2018-025367 (PMC6858131; doi:10.1136/bmjopen-2018-025367)
Supplement: Supplementary data [file bmjopen-2018-025367supp002.pdf]

Supplementary File B: supporting data

Explanation of observed activities:

'Front door' activity (initial assessment in emergency department, brain scan, and thrombolysis); Consultant-led ward rounds; Multi-Disciplinary Team (MDT) meeting catch ups (where staff update on patient status and discuss new admissions - only done in four of the HASUs); Nurse handover (at end of shift, nurses discuss patient status with nurses taking over); Bed meetings (where repatriation status of all patients is discussed); and Discharge (including discussions between HASU staff, external services, patients and carers).

Themes	Mechanisms/strategies
**1. Achieving consistent provision of 'front door' interventions at different times**	
1a. Adapting and extending roles	***Junior physicians - additional decision-making***<br>"When it comes to decision-making, of course thrombolysis calls will be calling the consultants. If it's not a thrombolysis call, most of the HASUs [hyperacute stroke units] allow us to take the decisions meaning to give to other specialty or ring them to your unit. And we take that in the middle of the night" (Junior doctor, H2)<br><br>"SHOs [senior house officers] are competent enough to do what is important, in other words exactly kind of, kind of tell the consultant exactly what the patient has from the history and examination which SHOs are very competent" (Junior doctor, H2)<br><br>***Nurse in charge - increased responsibility***<br>"The consultant stays until 5pm, the SHO until 9pm then the NIC [nurse in charge] will be the most senior staff. No HASU medical cover at night. If the nurse needs help, she will call the medical ward doctor and also the on call HASU consultant has access to telemedicine and will cover remotely" (observation, H5) "nurses who have been on day shift meet up with new nurses and they do bedside handovers, with the observation charts, and go through what the night schedule should entail." (OH [out of hours] evening observation, H2)<br><br>"Not just guiding the system, they are actually guiding, some good nurses actually, they actually hint when junior doctors are not sure what is going on. Like, is it a thrombolysis or not." (Junior doctor, H2)
1b. Creating continuities between day and night	**Processes - handovers, meetings**<br>"All therapy have a 8.45am handover from the nurse, therapy and dietician and psychology" (IH [in hours] observation, H6)
	**Designating staff to particular bays**<br><br>"Lately we've moved into a system where the medics are now being appointed to specific beds which I think has helped in us being able to direct which doctor to which family member so they can access that information rather than not necessarily being able to direct through the right person. So certainly in terms of any handover of information, at meetings it now seems to be more useful because they've got say six patients under their care and they're taking more onus and responsibility." (Occupational Therapist, H8)
	**Overlapping/extending roles**

Supplementary File B: supporting data

Themes	Mechanisms/strategies
	"SHO was supposed to leave a lot earlier (was here in the evening shift and stayed for handover) and there he is interrupted by the nurses and locum and he is typing up discharge documents. He seems more stressed now but happy to continue working until he has completed his jobs." (OH weekend observation, H1)<br><br>Initially, I was the one who did the working til' six to see if there was any benefit. […] I would often be able to see two patients. In that time from 4.30 to six and maybe discharge one or two, which made the next day, when the therapists came in, we could say those people are done you don't have to worry about them. (Occupational Therapist, H3)<br><br>"The SHO that was covering weekend and last night was at the MDT [multidisciplinary team] meeting and provided information on patients. Although not all staff knew all patients, there was always at least two members of staff that could provide updates on patients (MDT): "she was very confused on Sat"." (IH observation, H8)<br><br>"On the juniors' level, we have registrars, and I've changed that, so this was … used to be 9 to 5 and there was a handover at 5, and there was another handover at 9, so I've changed that. And there was also weekend coverage only by a neurology registrar, so I've changed that so it's now 9 to 9 stroke registrar coverage, as well as 9 to 9 on … 9 am to 9 pm on weekends." (Consultant physician, H3)
1c. Facilitator: Building relationships and trust	**Shared ethos and confidence to make decisions**<br>"It can get extremely busy and it can get very stressful, but I think um, everyone on the team is, is motivated and works hard, and um, I think there's, there are very good working relationships between, between all the staff on the Unit. It's a very enjoyable unit to work in, even when it's very busy." (Junior doctor, H8)<br><br>"You can feel the tension but you work in stroke because you really want to and you're dedicated to it so drive & motivation is a big part… you'll make it work because it's what needs to happen for your patients" (Occupational Therapist, H2)<br><br>"my seniors have high expectations I suppose and I think the doctors have quite high expectations of you know, I think I feel that rehab's quite a big part of this department so it makes me motivated, to try, you know as hard as possible. And I think that the whole team, team working aspect I think is important." (Physiotherapist H2)<br><br>"I think one of the joys of my job is, is my relationship with the nurses… I think I discovered that I need as a person and as a professional, I need to feel that my work matters." (Speech and Language Therapist, H8)
	**Effective relationships with other specialties (A&E, neuroradiology)**<br><br>"We have a, um, a weekly neuroradiology meeting where we discuss difficult scans in addition to looking at reports. And obviously they are just downstairs so we often pop down and say hello to them" (Consultant physician, H2)<br><br>"We work on very good terms with the radiology and I don't think we can function without that so yes they do prioritise we have, even when we get thrombolysis calls, we get plain CT [computerised tomography] head scans,

Supplementary File B: supporting data

Themes	Mechanisms/strategies
	we are actually very, they are very accessible, we walk into the reporting room and we can interrupt whatever they are doing and ask them to look at the scan quickly and see if there's any contraindication so very good working relationship and er we work very closely with them" (Junior doctor, H3)<br><br>"Sister said that A&E [accident & emergency] are becoming more aware of the urgency for stroke and so things improve." (IH observation, H8)<br><br>"They are very involved, I think that's what helps us with all our targets and everything on stroke care because Radiology, A&E… we have a thrombolysis meeting, every audit meeting every month… so everyone is really involved" (Senior nurse, H6)<br><br>"We don't ask them things, we tell them things […] we do everything ourselves and then we'll say to them, "okay this patient has had a stroke, I'm taking them to HASU," or, "this patient hasn't had a stroke, they've been handed to the medical team." (Stroke coordinator, H1)<br><br>"We've got a good relationship with the neuroradiology team. They are not able to give us quick enough reports for the Thrombolysis.[…] So the Stroke team, we do all the analysing, all the imaging before a Thrombolysis decision is made ourselves. And then it's later reviewed by radiology, in the next few hours afterwards […] I don't think any self-respecting, consultant should be making Thrombolysis decisions without themselves evaluating the imaging." (Consultant physician, H2)
1d. Facilitator: prioritisation of 'front door' interventions by staff	"HASU is quite a quick turnover, so we get a lot of patients, very interesting patients sometimes, and that kind of keeps the morale quite up, like, you know, we have thrombolysis calls, which is quite exciting" (Junior doctor, H8)<br><br>"I like the Hyper Acute side more, um, the Rehab side I worked for a year, year or two first and it's very good um, but I do prefer the faster paced Hyper Acute side better." (Nurse, H5)<br><br>"I really like to focus on you know early scanning, early admission to HASU, early specialist assessment by all of the members of the MDT, and you know I think for me early access to stroke unit care is probably the biggest thing that we've done in terms of stroke services. (Consultant physician, H7)
1e. Unintended consequences of adaptations	***Unintended consequence - Over-admission out of hours***<br>"Are they [registrars] maybe too cautious, and could they maybe be more brave in saying, 'No, I am sure this isn't a Stroke'? But I know they don't want to miss any strokes, so I think, you know, they have to be cautious." (Occupational therapist, H3)<br><br>"If I am a registrar in the middle of the night it is 50/50 of not too sure 100%. I'd rather bring them in, observe in the morning, get another opinion by a consultant and then think about what to do." (Junior doctor, H2)<br><br>"some of the registrars are excellent and have done lots of strokes and you may get a very junior registrar who I think, I think they'll always going to be safe… and provide safe care but in terms of efficiency… and there is something about experience that a consultant would add something in that respect." (Consultant physician, H8)

Supplementary File B: supporting data

Themes	Mechanisms/strategies
	"Overall, he [junior doctor] thinks it's a little hard to make the thrombolysis call because even though consultants have access to the scans at home, he doesn't know if he really wants to bother them when they're away and feels a little uncomfortable providing the appropriate information so that the appropriate decision can be made" (OH observation, H1)
**2. Explaining clinical interventions that displayed temporal variation**	
2a. Variations in medical, managerial and allied health professionals by time of day	**Consultant assessment**<br>Staffing reduces in the evening; only one ward round in the morning<br><br>"Matron talks to me again about how 5pm is a difficult time as the shifts end and families ask questions, patients get discharged and there aren't enough staff to sort it all out. She's changed the nurse rotas but can't do much about other professions." (IH observation, H5)<br><br>…now the workforce falls and there's only say 2 of us, a registrar and SHO, jobs that are not urgent at that point with regards to investigations and tests, it's come to 5 o'clock and a patient hasn't been transferred, well it is unlikely they are going to be later that evening (Junior Doctor, H4)<br><br>"It is difficult being NIC & having to go to thrombolysis calls. You can be in A&E all day and not know what's happening on the ward … Then when you come back to the ward everyone comes up to you for advice or questions. But I haven't been here all day and I don't know what's going on!" (OH evening observation, H6)<br><br>"X holds the bleep, which goes off at 8:35. nurse cringes: she is the only band 6 on for the evening, usually there are 2 band 6s. If there are 2, then one can go down when there is a bleep. But if there is only one, she doesn't feel she can go down. She and the nurse who had been head nurse in the ward for the day agree that 'we are responsible for the ward' and the bleep is secondary to this. A major factor in her decision is that the nurses who are on tonight are 'too junior' and 'too inexperienced' for her to leave them." (OH evening observation, H4)
2b. Variations in delivering therapist assessments	"We are staffed according to the standards. And one of our issues is we have seven day a week therapy, um, and we are not fully staffed to deliver seven day a week properly." (Consultant physician, H2)<br><br>"if you've turned up on the Tuesday morning, you'd be seen that day or within the, by a therapist within 24 hours. Whereas at the weekend, you're not being seen by an actual OT or a physio or a speech and language, so if you're nil by mouth on the Friday when you come in, say at half past four … you could technically be nil by mouth until Monday" (Speech and Language Therapist, H1)<br><br>"I think we're finding patients sometimes will get prioritised off, so they may not be seen for sort of ongoing therapy as much as we would like…" (Speech and Language Therapist, H6)<br><br>"speaking about therapy provision(just PT [physiotherapy]) on weekends> Yeah just 9 until 12 on the Saturday morning. When there's a bank holiday they shift it to a Sunday so that we are meeting the 72 hour window for the big national guidelines… but yeah that's the only input that we have over the weekend…. It can be quite difficult on Saturday, you are only here for 3

Themes	Mechanisms/strategies
	hours and if there's a lot of new patients because you don't necessarily get to see everyone… if you've got particularly heavy patients that come in or patients who have had quite severe strokes that are requiring more than 1 therapist to assess then it's quite difficult because you can't really, you are quite limited in terms of the assessments that you can do with them" (Physiotherapist, H5)<br><br>"The consultant acknowledges that the weekend is different to some of the patients. One patient can't be discharged yet because OT haven't assessed him, but otherwise looks well. The consultant suggests that he might have to be moved to the SU [stroke unit] to create space. Another patient was admitted the previous day, and wants to know what will happen and when she will start to regain speech and movement. Consultant: "it's quiet today, there will be a lot more activity tomorrow". He seems a bit apologetic and the visit is tokenistic - he can't offer her anything new. One patient gets even less interaction: "I'll update you again tomorrow"." (OH weekend observation, H5)<br><br>"SaLT [speech and language therapist] – down 1 member of staff – "very busy, 13 new admission and we're not working weekend so very busy". "They tried to start the model for the weekend work but there were no staff that wanted to do it. So they're looking at a different model now"" (IH observation, H8)<br><br>"That's the risk that you run, the quicker you try and get someone out and if you've not dealt with it fully, then you've got a high risk of readmission and a failed discharge" (Occupational Therapist, H1)<br><br>"Our main priority is patient safety, but we are pressured to discharge people, definitely." (Occupational Therapist, H1)<br><br>"As long as they're not needing an investigation for the Monday then they will go home, we will make referrals but obviously we can't follow them up until the Monday, because obviously with the community teams aren't working, if anyone needs a package of care restart or anything, then they'll have to stay until Monday as well." (Physiotherapist, H7)<br><br>"SSNAP [Sentinel Stroke National Audit Programme] coordinator said they have the backlog from the weekend, as is Monday today and they don't work at weekends. She is working at her desk at the nurses office and going through the patient list to collect live data ("my own ward round"). She said that the key indicators (SSNAP) are put together and then they average them with patient indicators and are adjusted by audit compliance (20% - speed of input and transfer). This she said is doctors related and doctors forget to fill it in." (IH observation, H8)<br><br>"You've got people off during the week so then it's hard sometimes to cover their caseload. […] I guess if we see patients at the weekend then they might not get therapy on the days they've had off" (Physiotherapist, H2)<br><br>"Therapists liaise directly with Nurse in Charge about who is priority to see; consultant sees patients and decides who to "step down", and defers discharge decisions to therapists […] therapists don't do rehab on the weekend (Weekend observation, H2)

Supplementary File B: supporting data

Themes	Mechanisms/strategies
2c. Factors influencing length of stay	**Factors within HASU/hospital**<br>"Using a lot of agency nursing staff recently as they currently have 23 B5 [band 5] vacancies. He says that the issue with agency staff is that they always say they can't do something so then it falls to the NIC to do it on top of everything else they need to do. For example the agency nurses will often refuse to put in NG [nasogastric] tubes or take bloods… "anything that has any amount of risk" because they are only there for 1 day and don't want anything to happen." (OH weekend observation H2)<br><br>"Lots of tests of movement and speech. "Nothing going to happen over the weekend" (consultant) but she can go home on Monday. Consultant wants a Doppler but he and the SHO discuss the difficult of getting this on the weekend. They agree that radiology treat weekend working as 'emergency' only whereas HASU tries to carry on normal working over the weekend (despite fewer medical staff). Patient discharge literally withheld by lack of therapists' input in this case." (OH weekend observation, H4)<br><br>"When NIC went away a few times, there was nobody at the main reception desk. At 10.16am the phone rang for a while, NIC came to answer from the bay that she was –with patient." (OH weekend observation, H3)<br><br>"At 10.10am nobody at the main desk. The phone was ringing and that is why the nurse from the bay where the porter was waiting, was not there as she was on the phone at reception. She said that in bay two they are very busy, her bay is not full, but there a couple of admissions yesterday" (OH weekend observation, H7)<br><br>"From the handover it was apparent that some patients who were admitted at the weekend did not have all the tests done or therapy assessment. Nurse: 'he's waiting for carotid doppler'" (IH post-weekend observation, H1)<br><br>"Patient with slurred speech; no SaLT on weekend so SHO asks patient if he wants to go home & wait for SaLT in community (there will be a wait for one) or stay in hospital & wait until Monday to see one (patient decides to go home)" (OH weekend observation, H2)<br><br>**Factors beyond HASU**<br>"Social Services aren't open at the weekends, so it's, until that changes, it won't be the same care, there's always going to be a block" (Physiotherapist, H8)<br><br>"I think one of the problems is that many of these stroke rehab wards are … out of hours they're covered by the medical on call team, so basically, for cardiac arrest, severe medical problems and things like that, so that if you do transfer them, on a Saturday or Sunday, they don't really get seen in any, detailed way until the Monday, so there is a little concern about the safety of that … so […] we would keep that … hold that transfer until the Monday." (Consultant physician, H8)<br><br>"We still can't discharge people on Thursdays because social services are closed from four o'clock on Friday. The community teams are not working, you know, so we can be here but we still can't get people home if they need any of that stuff." (Occupational therapist, H3)<br><br>"A lot of the issues I think are external to the Trust and that is probably our biggest bugbear when patients are awaiting nursing home placements and

Supplementary File B: supporting data

Themes	Mechanisms/strategies
	things like that. They're not actually benefitting anymore from an acute stroke bed, but they are blocking a bed in an acute stroke Trust because there is nowhere for them to go." (Stroke coordinator, H4)<br><br>"The ESDs [early supported discharge(s)] have been set up to mimic a stroke unit at home, but their capacity changes, so before we can discharge someone for ESD, we have to phone them, make sure they've got capacity, they run through a checklist with us, we have to make sure the equipment is delivered and so forth. And you couldn't do any of that at weekends, so we instruct the therapists that work the weekend, if someone needs to go home with ESD, they have, they've got to stay in until Monday anyway." (Physiotherapist, H8)<br><br>"Now they've kind of been set up, the issues, issues we have is every borough has, borough has a different criteria" (Physiotherapist, H5)<br><br>"We work with five local boroughs, that's five different ESD teams, plus all the others around that, so we, we work closely with five ESDs and we work frequently with probably about another five, so it's quite, so that's why we have to try hard (laughs) to keep up the communication" (Physiotherapist, H8)<br><br>"The only delay to discharges here are the patients waiting for nursing homes. But that's the case across the whole hospital. And that is because there aren't enough services in the community, as everybody knows this is not news. So because of that at any one time we can have anything between 6 and 11, I think 11 was our maximum people waiting for nursing homes. It's not their fault, it's not their family's fault, it's not anybody's fault, it's a simple case of there is nowhere for them to go." (Stroke coordinator, H1)<br><br>"NIC: Supports the nurse, also looks after a bay and also has the bleep and had to deal with the repatriation (notes left for her by the repat/stroke coordinator)." (OH weekend observation, H3)<br><br>"Repatriation nurse: Monday is always horrendous lots of outliers – 11 outliers, just because they don't repatriate the same at the weekend and don't process the same way at the weekends at the receiving end. Some SUs are at red alert and they prioritise their own patients in A&E. Over the weekend cannot make discharge decisions because the therapists are not available." (IH post-weekend observation, H8)<br><br>"There is one patient waiting repatriation but there are issues with the receiving hospital as they have no free beds. Lead consultant to nurse: so we haven't heard from [hospital] yet?, cause we referred him, what 10 days ago!?" (IH observation, H2)<br><br>"We will make referrals but obviously we can't follow them up until the Monday, because obviously with the community teams aren't working, if anyone needs a package of care restart or anything, then they'll have to stay until Monday as well." (Physiotherapist, H7)<br><br>"We're having to take on jobs that we wouldn't otherwise have to do if those patients were flowing through to their local SUs more quickly." (Speech and Language Therapist, H7)

Supplementary File B: supporting data

Themes	Mechanisms/strategies
	One patient can't be discharged yet because Occupational Therapist hasn't assessed him, but otherwise looks well. The consultant suggests that he might have to be moved to the SU to create space. (OH Weekend observation, H5)<br><br>"We do have some delays with repats [repatriations] but we would see them here in the meantime, and then they'd be onto, transferred onto our Stroke Unit until a bed becomes available at, at the, the repat hospital." (Physiotherapist, H5)<br><br>"I think that would be brilliant, that would be one of my biggest things, having a social worker present on the board round. It would free therapists up to do therapy work, to facilitate discharge at a timely manner would be much more efficient and a smooth pathway" (Occupational therapist, H1)<br><br>"One patient can't be discharged yet because OT haven't assessed him, but otherwise looks well. […] Another patient was admitted the previous day, and wants to know what will happen and when she will start to regain speech and movement. Consultant: "it's quiet today. There will be a lot more activity tomorrow". He seems a bit apologetic and the visit is tokenistic - he can't offer her anything new." (OH Weekend observation, H5)<br><br>"Social services are closed from four o'clock on Friday. The community teams are not working, you know, so we can be here but we still can't get people home if they need any of that stuff." (Occupational therapist, H3)
